# VESALIUS keeps revolutionizing medicine: the case of evolocumab in primary prevention

**DOI:** 10.1093/ehjcvp/pvaf083

**Published:** 2025-11-21

**Authors:** Mattia Galli, Sebastiano Sciarretta, Filippo Crea

**Affiliations:** Department of Medical-Surgical Sciences and Biotechnologies, Sapienza University of Rome, Corso della Repubblica, 79, Latina 04100, Italy; Maria Cecilia Hospital, GVM Care and Research, Cotignola 48033, Italy; Department of Medical-Surgical Sciences and Biotechnologies, Sapienza University of Rome, Corso della Repubblica, 79, Latina 04100, Italy; IRCCS NeuroMed, Pozzilli, Italy; Center of Excellence of Cardiovascular Sciences, Ospedale Isola Tiberina—Gemelli Isola, Rome, Italy; Catholic University of the Sacred Heart, Rome, Italy

Lowering low-density lipoprotein (LDL) cholesterol reduces cardiovascular events in both primary and secondary prevention.^1-4^ Within the spectrum of ‘residual lipid risk’, several other markers have emerged as important contributors, including non-high-density lipoprotein (HDL) cholesterol, apolipoprotein B (ApoB), and lipoprotein(a) [Lp(a)].^1^

Pro-protein convertase subtilisin/kexin type 9 (PCSK9) inhibitors have demonstrated efficacy in reducing ischaemic events among patients with prior myocardial infarction or stroke.^3,4^ Notably, these agents also lower Lp(a) levels by 19%–27%, an effect not observed with statins or ezetimibe.^1^

The placebo-controlled, double-blind, VESALIUS-CV trial addressed a critical and previously unanswered question: can PCSK9 inhibition reduce major adverse cardiovascular events (MACE) in patients without prior myocardial infarction or stroke? In this landmark study, 12 257 uneventful patients with atherosclerosis or diabetes and at least one of the following—LDL cholesterol ≥90 mg/dL, non-HDL cholesterol ≥120 mg/dL, or ApoB ≥80 mg/dL—while receiving optimized lipid-lowering therapy for at least 2 weeks, were randomized to evolocumab 140 mg every 2 weeks or placebo.^5^ The cohort included 43% women, 69% Europeans, and 59% with diabetes; 67% had documented atherosclerosis (45% coronary, 10% cerebrovascular, and 17% peripheral). Overall, 68% of patients were receiving high-intensity statin therapy, and 19% were treated with ezetimibe. Baseline LDL and ApoB levels were 122 and 102 mg/dL, respectively.^5^ After a median follow-up of 4.6 years, evolocumab significantly reduced the risk of 3-point MACE (coronary death, myocardial infarction, or ischaemic stroke) by 25% and 4-point MACE (including ischaemia-driven revascularization) by 19%. Although hierarchical testing precluded statistical confirmation, reductions of 21% in cardiovascular mortality and 20% in all-cause mortality were observed. The median LDL cholesterol achieved at 48 weeks was 45 mg/dL with evolocumab vs. 109 mg/dL in the placebo group.^5^

However, it remains uncertain whether achieving such LDL targets through other lipid-lowering drugs would yield equivalent outcomes.^6^ This uncertainty is relevant not only for understanding the potential class effect of PCSK9 inhibitors, but also for evaluating the cost-effectiveness of different lipid-lowering strategies.

This major advance parallels the breakthroughs achieved with GLP-1 receptor agonists and SGLT2 inhibitors, which have reshaped prevention across primary and secondary settings.^1,7^ As only ∼15% of patients received these agents, whether the benefit of intensive lipid lowering is additive remains uncertain. Remaining challenges include refining the identification of high risk patients and ensuring the long-term affordability and sustainability of these life-saving interventions.

## Data Availability

No research data have been used for this manuscript.
